# Rationale of Probiotic Supplementation during Pregnancy and Neonatal Period

**DOI:** 10.3390/nu10111693

**Published:** 2018-11-06

**Authors:** Maria Elisabetta Baldassarre, Valentina Palladino, Anna Amoruso, Serena Pindinelli, Paola Mastromarino, Margherita Fanelli, Antonio Di Mauro, Nicola Laforgia

**Affiliations:** 1 Neonatology and Neonatal Intensive Care Unit, Department of Biomedical Science and Human Oncology, “Aldo Moro” University of Bari, P.zza Giulio Cesare 11, 70124 Bari, Italy; valentinapalladino@hotmail.it (V.P.); amorusoanna@hotmail.it (A.A.); stefsere@alice.it (S.P.); antonio.dimauro@uniba.it (A.D.M.); nicola.laforgia@uniba.it (N.L.); 2 Section of Microbiology, Department of Public Health and Infectious Diseases, Sapienza University, Piazzale Aldo Moro 5, 00185 Rome, Italy; paola.mastromarino@uniroma1.it; 3 Department of Interdisciplinary Medicine, “Aldo Moro” University of Bari, 70100 Bari, Italy; margherita.fanelli@uniba.it

**Keywords:** “Probiotics”[Mesh], “Pregnancy”[Mesh], “Infant, Newborn”[Mesh]

## Abstract

Probiotics are living microorganisms that confer a health benefit when administered in adequate amounts. It has been speculated that probiotics supplementation during pregnancy and in the neonatal period might reduce some maternal and neonatal adverse outcomes. In this narrative review, we describe the rationale behind probiotic supplementation and its possible role in preventing preterm delivery, perinatal infections, functional gastrointestinal diseases, and atopic disorders during early life.

## 1. Introduction

Gut microbiota is a heterogeneous microbial community that includes 10^14^ microorganisms comprising predominantly bacteria, but also viruses, archaeans, and protozoa, and is considered as a super-organ that dynamically interacts with the host in a mutual relationship [1,2]. Gut microbiota plays a significant role in human immunology, nutrition, and pathological processes. Despite inter-individual variability, in adults, 80% of gut microbiota is composed of three dominant phyla: *Bacteroidetes*, *Firmicutes,* and *Actinobacteria* [3]. The final composition of the intestinal microbiota is influenced by multiple factors such as genetic heritage, type of delivery, mode of feeding, administration of probiotics or antibiotics, stress, and infections [4]. The neonatal microbiota is highly different compared to the adult one, since the first is characterized by rapid changes [5]. At birth, the newborn is exposed to a set of bacteria including *staphylococci*, *enterobacteria,* and *enterococci* that immediately colonize the gastrointestinal tract. In the first days of life, the gut is inhabited mainly by *Bifidobacterium, Lactobacillus*, *Clostridium,* and *Bacteroides*. From one to five months of life, the population of the gastrointestinal tract consists of *Bifidobacteriales*, *Lactobacillales,* and *Clostridiales*. At one year of age, the microbiota is similar to the adult one [6,7]

Traditionally, babies have been considered sterile in utero while microbes colonize their gut during delivery and after the birth [8]. Several studies suggest that the placenta and amniotic fluid are involved in this process. In fact, the fetus incorporates an initial microbiome before birth [9,10]. Placental microbiome composition has been recently characterized, and includes non-pathogenic strains of *Bacteroidetes Firmicutes*, *Fusobacteria*, *Proteobacteria,* and *Tenericutes* [11]. During pregnancy, the ingestion of bacteria present in the amniotic fluid influences the foetal gut microbiome. Further, maternal microorganisms are present in the meconium and in the cord blood [12,13] in the total absence of chorioamnionitis. 

The microbiota colonizes the host before birth and matures definitively during the twelve months following delivery [14]. During this moment, the fetus comes into contact with maternal vaginal bacteria that immediately reach the newborn gastrointestinal tract. The gut of infants born vaginally are colonized prevalently with *Bifidobacterium* and *Streptococcus*. In contrast, caesarean delivery is associated with a decrease of *Bifidobacteria,* while *Clostridium* and *Bacteroides* prevail [15,16,17]. There is some evidence that *Bifidobacteria* influence the development of very common allergic disorders such as atopic eczema and asthma [18,19]. Additionally, cesarean sections, especially as elective procedures, seem to represent a risk factor for autoimmunity and metabolic disorders [20,21]. Moreover, *bifidobacteria* are the most represented bacteria in the gastrointestinal tract of healthy infants. Beside the type of delivery, other factors affect microbial colonization in newborns. The abuse of antibiotics during pregnancy or after birth seems to reduce the number of *bifidobacteria* [22]. Schumann at al. have recently demonstrated a severe decrease of intestinal aerobic and anaerobic bacteria in rats treated with daily intragastric gavage of amoxicillin [23]. The gestational age at the birth is one of main factors that delineates the profile of gut microbiota. In fact, preterm newborns, in comparison to term births, have higher rates of anaerobic bacterial colonization, in particular *Enterobacteriaceae* [24] and *Enterococcaceae* [25,26]. During a premature delivery, it is not guaranteed that close contact with the vaginal mucosa and a smaller amount of bacteria are ingested. Additionally, in neonatal intensive care units, the wide use of antibiotics contributes to reduced growth indexes of gut bacteria, creating a restricted microbial population [27]. Abnormal vaginal microbiota or active bacterial infection during pregnancy alter the acquisition of neonatal flora promoting preterm delivery [28]. The presence of pathogenic bacteria in the amniotic fluid activates the innate immune response, and the production of prostaglandins increases uterine contractility, promoting premature birth [29].

Moreover, breastfeeding is another important determining factor in establishing the gut microbiome, and is a source of short- and long-term health benefits for the child. In the short term, it has been observed that it decreases the risk of infections, diarrhoea, type-1 diabetes, and necrotizing enterocolitis; while the long term benefits of breastfeeding include protection from the development of diseases like type-2 diabetes, inflammatory bowel disease, and obesity [30]. Breast milk contains fats, proteins, cytokines, enzymes, antibodies, and nutrients that influence the growth of the child and the development of his/her immune system [31]. Other components are antimicrobial agents like lactoferrin, lysozyme, peroxidase, defensins, IgAs, and oligosaccharides. The rich composition of human milk provides passive immunoprotection against infections and inflammation [32]. 

Among these components, lactoferrin is an important protein in breast milk, mostly in colostrum, and is involved in the regulation of the immune system and inflammatory response. A recent study suggests that during breastfeeding, lactoferrin is transferred to the intestine of the newborn. The fecal concentration of this protein progressively increases in the first month after birth, promoting the growth and differentiation of the immature intestine. Therefore, lactoferrin seems to promote the proliferation of enterocytes and closure of enteric gap junctions regulating the postnatal intestinal development [33]. Finally, lactoferrin is considered as a growth promoter for *bifidobacteria*, the predominant beneficial microorganism of human gut [34].

Furthermore, there is accumulating evidence that human milk is not sterile, but contains maternally-delivered bacteria, i.e., mainly lactobacilli and bifidobacteria. The number of bacteria ingested by an infant per 800 mL of milk consumed daily is estimated at 1 × 10^5^–1 × 10^7^ [35].

Microorganisms present in the breast milk are transferred from the mother’s intestine to the mammary gland through the lymphatic system by dendritic cells by openings in the tight junctions of the intestinal epithelium [36]. The bacteria contained in the breast milk affect the composition of the gut microbiota in infants, and they could protect against infectious diseases and promote the maturation of the immune system. [37,38]. Additionally, other factors in breast milk including oligosaccharides, s-IgA, and lactoferrin influence the proliferation of healthy microbiota [7,39]. Intestinal bacteria stimulate endogenous production of s-IgA [40], activation of T regulatory cells [41,42] and anti-inflammation response [43,44]. Therefore, appropriate gut colonization through breastfeeding is involved in the correct development of immune system and in prevention of diseases.

Gastrointestinal flora composition differs mostly in breast- and formula-fed infants. Several studies conducted on stool samples of newborns have shown that bifidobacteria are present in the flora of both groups, but their number is higher in breastfed infants in comparison to formula-fed infants; instead, the number of *E. coli* and *Bacteroides* is higher in formula-fed infants [6]. These differences remain, even after breastfeeding is discontinued [7].

Current evidence supports a link between the activity and composition of the gut microbiota and human health and disease. The correct development of gut microbiota composition affects many organs, including neural, immune, and gastrointestinal systems. The gut microbiota composition is altered in many diseases, like disorders of the gut-brain axis [45], immune and gastrointestinal disorders [46,47], and allergic diseases [48]. The potential modulation of the gut microbiota through the administration of probiotics is very prominent in the prevention of human diseases starting from pregnancy.

## 2. Methods

An exhaustive search for eligible studies was performed in PubMed, Embase, Medline, Cochrane library and Web of Science database.

The following subject MeSH headings were used: “Probiotics”[Mesh], “Pregnancy”[Mesh], “Lactation”[Mesh], “Breast Feeding”[Mesh], “Premature Birth”[Mesh], “Infection”[Mesh], “Gastrointestinal Diseases”[Mesh]. Furthermore, free text for “allergy”, “atopy”, “gut-brain axis”, and proper Boolean operators “AND” “OR” were also included to be as comprehensive as possible. Additional studies were sought using references in articles retrieved from searches.

Search limits were set for RCT, involving only human subjects, and published between October 2008 and October 2018. The review was limited to studies written in English.

## 3. Role of Probiotics Administration in the Prevention of Infection and Preterm Delivery during Pregnancy

Vaginal microbiota alterations and infections during pregnancy lead to a greater possibility of preterm delivery; this is related to the development of neonatal infections, sepsis, and necrotising entercocolitis. The use of probiotics seems to modulate the composition of vaginal microflora. Vitali et al. have conducted a pilot, non-randomized, controlled, and perspective study that demonstrated the influence on the vaginal microbiota of pregnant women of dietary supplementation with a probiotic mixture containing *L. paracasei* DSM 24733, *L. plantarum* DSM 24730, *L. acidophilus* DSM 24735, and *L. delbrueckii* subsp. *bulgaricus* DSM 24734), three strains of bifidobacteria (*B. longum* DSM 24736, *B. breve* DSM 24732, and *B. infantis* DSM 24737), and one strain of *Streptococcus thermophilus* DSM 24731, produced at Danisco-Dupont, WI, USA and currently sold in Continental Europe and USA under the brand Vivomixx^®^ and Visbiome^®^, respectively. The characterization of vaginal bacteria in women supplemented with this multistrain probiotic showed an increase of *bifidobacteria* and a reduction of *Atopobium vaginae,* resulting in the prevention of bacterial vaginosis. Furthermore, IL-4 and IL-10 levels are influenced by alteration in the vaginal microbial environment. The decline of cytokines involved in the antiphlogistic process were noticed in a control women group that did not consume the probiotic mixture [49]. In contrast, Gille et al. in a recent trial demonstrated that the supplementation with *Lactobacillus rhamnosus GR-1* and *L reuteri RC-14* for two months during pregnancy does not improve the normal composition of vaginal microbiota compared to the placebo group [50].

Group B Streptococcal (GBS) vaginal colonization is considered a principal cause of neonatal sepsis, pneumonia, and meningitis [51]. The Centres for Disease Control and Prevention (CDC) suggest parenteral antibiotic administration during delivery as preventive therapy for women diagnosed with GBS at 35 and 37 weeks of gestation [52]. The possible impact of probiotic administration on prevention of infections during pregnancy has been investigated.

Recently, Olsen at al. performed a randomized pilot study to determine a potential causal relationship between probiotic administration during pregnancy and vaginal Group B Streptococcal (GBS) colonization. There was no significant difference in the incidence of GBS vaginal infections between the women supplemented with probiotics and the control group. However, a greater proportion of commensal bacteria was found in pregnant women who had used probiotics [53]. Besides Ho M. et al. conducted a randomized controlled trial to examine the effect of the oral administration of *Lactobacillus reuteri RC-14* and *Lactobacillus rhamnosus GR-1* in pregnant women with a vaginal and rectal GBS colonization. Compared to the placebo group, women treated with probiotics had significantly-reduced rectal and vaginal GBS colonization rates [54].

Bacterial vaginosis increases the risk of spontaneous preterm delivery and neonatal complications [55]. Few studies have tested the efficacy of probiotics in the prevention of preterm births. A prospective cohort study recently showed that the administration of a milk supplemented with probiotics during pregnancy reduced preeclampsia and preterm delivery risk [56]. Furthermore, a randomised controlled trial tested the early administration effect of *Lactobacillus rhamnosus GR-1* and *Lactobacillus reuteri RC-14* in women during gestation affected by low/intermadiate grade of vaginosis, to have a reduce premature delivery risk [57].

In a randomized clinical trial, the use of a yoghurt that contained *Lactobacillus bulgaris*, *Streptococcus thermophilus*, *Probiotic lactobacillus*, and *Bifidobacterium lactis* has been investigated in pregnant women in the treatment of bacterial vaginosis versus the use of clindamycin. Compared to the use of clindamycin, the administration of probiotics has a significant effect only on the reduction of vaginal pH, which seems to be associated with a lower risk of preterm delivery [58]. Therefore, there is no determinant evidence from clinical trials that confirms role of probiotics in the prevention of preterm delivery (Table 1). A recent metanalysis including 21 studies confirms that there is no evidence that the administration of probiotics in pregnant women reduces the risk of preterm delivery [59].

The potential role of probiotics in the prevention of infections during pregnancy and in preterm infants remains unclear, and requires further research.

## 4. Role of Probiotics Administration in the Prevention of Allergic Diseases

At birth, the lymphoid system of the newborn is not yet mature and Th1 response is inhibited. Therefore, it is necessary that the immune system clear the gap between Th1 and Th2 response. Microbiota has a crucial role during this critical phase [60]. There is a relationship between gut microbiome patterns and the potential role of modulation of innate immune signaling in the prevention of allergic diseases. West et al. have shown that after the birth, the maturation of the intestinal microbiota influences the innate immune response and the development of atopic eczema. This study suggests that alteration of gut microbial population increases the risk of the development of atopic-eczema due to a lack of modulation on inflammatory cytokines mediated by the microbiome [61]. Moreover, antibiotics, caesarean section, and infant formula are factors that modify microbiome composition and are related to the development of allergic diseases [62].

Probiotic supplementation to mothers during breastfeeding positively influences the microbial composition of breast milk and positively modulates the neonatal immune system mainly through the regulation of both Th1- and Th2-type response and by the stimulation of tolerance [63]. It has been observed that administration to mothers of a probiotic mixture containing *L. paracasei* DSM 24733, *L. plantarum* DSM 24730, *L. acidophilus* DSM 24735, and *L. delbrueckii* subsp. *bulgaricus* DSM 24734), three strains of *bifidobacteria* (*B. longum* DSM 24736, *B. breve* DSM 24732, and *B. infantis* DSM 24737), and one strain of *Streptococcus thermophilus* DSM 24731 (Danisco-Dupont, WI, USA and currently sold in Continental Europe and USA under the brand Vivomixx^®^ and Visbiome^®^, respectively) resulted in an increase of lactobacilli and bifidobacteria in both colostrum and mature milk [7]. This probiotic mixture seems to play a key role in the regulation of the immune response which is influenced by the type of delivery; by comparing women with vaginal delivery under probiotics or placebo, an increase in bifidobacteria and lactobacilli was observed in the colostra and mature milk in the first group; no difference, however, was observed between the two groups of women who underwent caesarean section [63]. Maternal probiotic supplementation before and after delivery seems to prevent atopic eczema in children (Table 2), but further studies are requested to confirm this relation with other allergic disorders.

In a randomized, double-blind trial, women were given probiotic milk or placebo from the 36th week of gestational age to 3rd month after delivery during breastfeeding. At 2 years of age, all infants were tested for atopic dermatitis, asthma, and other allergic diseases. The study demonstrates that administration of probiotics to mothers during pregnancy decreases the incidence of atopic dermatitis, but has no effect on asthma [64]. Enomoto et al., in an open trial, confirmed that the administration of a combination of Bifidobacteria from 1 month before delivery to mothers and 6 months after birth to babies significantly reduced the incidence of cutaneous allergic diseases (eczema/atopic dermatitis). Moreover, women in the study group exhibit lower fecal Proteobacteria concentrations; this is related to higher children fecal concentration of Bacterioidetes at 4 months of age [65].

In contast, a recent randomized placebo-controlled trial demonstrated that the administration of *Lactobacillus rhamnosus* HN001 to pregnant woman before and after delivery during breastfeeding seems not to prevent the development of infant eczema, wheeze, and atopic sensitization during the first year of life [66]. In addition, another clinical trial shows that the supplementation of *Lactobacillus* GG in women from the 6th month of pregnancy and after birth (to mothers during breastfeeding or to infants for 6 months) does not reduce the incidence of developing allergic diseases in children followed up to 36 months of age [67].

Rautava et al. found that maternal supplementation of a mixture of probiotics 2 months before delivery and 2 months after lactation reduces the risk of developing eczema in infants in the first 2 years of life [68]. Furthermore, Kim et al. prove that the maternal supplementation of *Bifidobacterium bifidum*, *B. lactis,* and *Lactobacillus acidophilus* 4–8 weeks before delivery and until 6 months after the birth reduces the prevalence of eczema in the first year of life in children [69].

In a pAnda study, a mixture of probiotics (*Bifidobacterium bifidum*, *Bifidobacterium lactis*, and *Lactococcus lactis*) administrated to mothers before delivery and to infants for the first year of life reduced the incidence of eczema during the first 3 months of life compared to placebo group [70].

Simpson et al. have confirmed the long-term protective effect on the baby of probiotic maternal administration: children assessed at 6 years of age have a lower incidence of the development of atopic dermatitis, while this has not been confirmed for other allergic diseases [71]. Therefore, several studies have confirmed the role of probiotics in the early prevention of eczema in children, and these benefits were shown to persist over time. A recent randomized, controlled study (Probiotics in the Prevention of Allergy among Children in Trondheim, ProPACT) in part explains how perinatal maternal probiotics supplementation can play a protective role in the development of atopic dermatitis. In this study T-regs, Th-1, Th-2, and Th-17 lymphocyte or the Th-1/Th-2 ratio seem not to be influenced by a mixture of LGG, La-5, and Bb-12, but this reduces the proportion of Th-22 number [72].

The World Allergy Organization (WAO) convened a guideline panel to develop evidence-based recommendations about the use of probiotics in the prevention of allergies. The WAO guideline panel suggests:

- that by using probiotics in pregnant women at high risk for allergy in their children, there is a net benefit resulting primarily from prevention of eczema (conditional recommendation, very low-quality evidence).
- that by using probiotics in women who breastfeed infants at high risk of developing allergy, there is a net benefit resulting primarily from prevention of eczema (conditional recommendation, very low-quality evidence).
- using probiotics in infants at high risk of developing allergies, because there is a net benefit resulting primarily from prevention of eczema (conditional recommendation, very low-quality evidence).

Currently-available evidence does not indicate that probiotic supplementation reduces the risk of developing allergies in children, but there is a net benefit primarily in the prevention of eczema [73].

## 5. Brain-Gut Microbiota Axis

The microbiota plays an important role in the interaction between the brain and the enteric nervous system, known as the “brain-gut microbiota axis”. Thanks to this axis, information from the Central nervous system can influence the motor, secretory, and sensitive functions of the gastrointestinal tract; conversely, visceral signals from the gut can modulate brain activity, mood, and behavior [7]. The gut-brain dialogue involves neuro-immuno-endocrine mediators [74].

It is believed that alterations in the microbiota gut-brain axis are associated with the onset of irritable bowel syndrome (IBS) or functional gastrointestinal (GI) disorders [75], and might be implicated in autism spectrum disorders (ASDs) [76,77,78], anxiety-depressive behaviors [76,79,80,81], and chronic pain [82]. However, the sites, the pathways and molecular mechanisms responsible for these alterations must be better defined. 

Different models have been used to define the gut brain axis (GBA), including gut microbial perturbation by antibiotics and probiotics, fecal microbial transplantation, and mice who lived without any exposure to microorganisms (germ-free, GF) [83]. GF animals were born by Caesarean section and lived in aseptic conditions.

There is supporting evidence that metabolites derived from maternal gut microbiome modulate the neurotransmitter, synaptic, and neurotrophic signaling systems, thus influencing fetal brain development [84]. Petterson et al. underline that “healthy” intestinal microbiota is crucial for the programming of mammalian neurodevelopment and later adult behavior. This study suggests that early microbial colonization influences the expression of synaptic-related proteins (e.g., synaptophysin, which is an indicator of synaptogenesis), signaling pathways, and neurotransmitter turnover, that could modulate the synaptic transmission influencing motor control and emotional behavior in adults [85].

It has also been reported that maternal separation (MS) in rodents induced dysbiosis and brain and behavioral changes [86]. MS triggers depression and anxiety-like behavior [87,88], hyper-responsiveness of the HPA axis [89], increased intestinal permeability [90,91,92], and visceral hypersensitivity [93].

Maternal stress and MS might be implicated with modifications of gut brain axis [94,95] and probiotics seem to improve those gut and brain changes caused by perinatal stressors [96,97,98]. In different studies, it was observed that postnatal microbial colonization regulates an adequate HPA response to stress [99] and the hippocampal serotoninergic system [100]. It was also described a decrease in depression and anxiety-like behavior after the administration of oral probiotics in mice [96,101,102] and humans [103,104] with normal gut microbiota.

Furthermore, the short-chain fatty acids (SCFA s) generated by the colonic microbiota represent a significant source of energy for the gastrointestinal cells. In this regard, colonocytes from germ-free C57BL/6 rodents showed lower energy statuses than normally-raised mice [105].

Additionally, the loss of intestinal-generated SCFAs induces metabolic changes that may affect neurodevelopment and alter mechanisms associated with feeding behavior and metabolism [83].

Indeed, recent studies compare ingestive behavior between mice with gut microbial composition and GF, suggesting that gut microbioma modulate feeding behavior. GF mice showed lower blood levels of leptin and ghrelin, and a higher inclination for lipids, justified by the increased expression of oral receptors for fats and a decreased one for gut fatty-acid receptors [106].

Furthermore, it was observed that *bifidobacterium B. longum 1714* improves cognition in mice [107]. Additionally, the administration of a probiotic mixture containing *L. paracasei* DSM 24733, *L. plantarum* DSM 24730, *L. acidophilus* DSM 24735, and *L. delbrueckii* subsp. *bulgaricus* DSM 24734), three strains of bifidobacteria (*B. longum* DSM 24736, *B. breve* DSM 24732, and *B. infantis* DSM 24737), and one strain of *Streptococcus thermophilus* DSM 24731 (Danisco-Dupont, WI, USA, currently sold in Continental Europe and USA under the brand Vivomixx^®^ and Visbiome^®^, respectively) to aged animals induced a reduction of the age-related attenuation of LTP through modifications of the gut microbiota [108]. These results are interesting but translational studies in humans are necessary.

## 6. Probiotics and Functional Gastrointestinal Disorders

Probiotics have an important role in the maturation and health of the intestinal tract. The composition of gut microbiota is involved in the development of gastrointestinal functions, particularly in the gut-brain axis, and participates in emitting and receiving signals to and from the brain [109]. This connection occurs with different mechanisms: through the release of cytokines and chemokine (immune pathway), through neural pathways, and through the production of intestinal neuroendocrine factors (endocrine pathway) [6]. Therefore, through changes of the microbiota, bidirectional relationships between the gut and brain are modified, thus influencing the pathogenesis of functional gastrointestinal disorders. The most common diseases in early life are infantile colic, a benign and functional gastrointestinal disorder that affects around 20% of young infants. Colic is associated with parental frustration and anxiety, and resolves spontaneously after the first three to four months of life. Although infantile colic is a self-resolving condition, it implies long-term effects on a child’s behavior, sleep, and allergies [110].

According to the Rome IV criteria for functional gastrointestinal disorders, infantile colic is diagnosed in infants younger than 4 months of age if the following symptoms occur: paroxysms of irritability, fussing or crying that starts and stops without obvious cause; episodes lasting 3 or more hours per day and occurring at least 3 days per week for at least 1 week; and no failure to thrive [111].

Infantile colic presents a multifactorial aetiology, but the cause remains unclear. However, some causative mechanisms have been suggested like behavioral, food allergies and hypersensitivities, immaturity of gut function, and dysmotility [112].

An extensive number of possible factors have been hypothesized, like increased painful intestinal contractions, lactose intolerance, food hypersensitivity, gas, parental misinterpretation of the normal crying pattern, and altered gut microbiota (dysbiosis). In the management of infantile colic, many therapies are used, such as dietary, pharmacological, and behavioral interventions. However, data on their effectiveness are limited. Dysbiosis may play an important role in the pathogenesis of infantile colic, and gut microbiota modification with probiotics can have advantages on the management of infantile colic [113].

Partty et al. have conducted a randomized, double-blind, prospective study based on the administration of *L*. *rhamnosus* GG (ATCC 53103) or placebo to mothers daily for 4 weeks before expected delivery, and subsequently to the child or the mother, if breast-feeding, for 6 months, to evaluate the influence on the appearance of functional gastrointestinal disorders.

They hypothesized that colic crying, typical of the perinatal period, was associated with functional gastrointestinal disorders later in childhood.

Their 13-year follow-up study showed that administration of *L. rhamnosus* GG (ATCC 53103) does not affect the appearance of functional gastrointestinal disorders later in childhood, but suggests that different probiotics or probiotic combinations may be needed [114]. (Table 3).

It has been shown that probiotic administration to women during pregnancy and lactation can change the composition of breast milk, and consequently, its immunomodulatory molecular composition, bestowing benefits on the child in the form of reduced instances of gastrointestinal disorders. 

A study conducted in 2013 showed an increase in breast milk of anti-inflammatory molecules such as TGF-B and IL-10 in supplemented mothers compared to the control group. Indeed, maternal probiotic supplementation leads to an increase of the TGF-B, which stimulates gut maturity, influencing IgA production and oral tolerance induction, and that seems to improve gastrointestinal functional symptoms in infants [115].

In a recent study, Baldassarre et al. have demonstrated that the use of a probiotic mixture (*L. paracasei* DSM 24733, *L. plantarum* DSM 24730, *L. acidophilus* DSM 24735, and *L. delbrueckii* subsp. *bulgaricus* DSM 24734), three strains of bifidobacteria (*B. longum* DSM 24736, *B. breve* DSM 24732, and *B. infantis* DSM 24737), and one strain of *Streptococcus thermophilus* DSM 24731, Vivomixx^®^, Danisco-Dupont, WI, USA) appears safe and reduces inconsolable crying in exclusively breastfed infants with infantile colic [116]. 

Many studies have also been conducted on the prophylactic use of probiotics in the first months of life to treat breastfed infants with colic. In particular, benefits of the use of *Lactobacillus reuteri* on functional gastrointestinal disorders have been studied.

Gutiérrez-Castrellón et al. [117] have demonstrated the superiority of the use of *L. reuteri DSM 17938* with a dose of 10^8^ CFU/day for 21 to 28 days to significantly reduce the duration of crying episodes during the day.

Recently, Indrio et al. [118] have suggested that oral supplementation of *L. reuteri,* for the first three months of life, not only reduces the probability of colic episodes and other functional gastrointestinal disorders, like gastroesophageal reflux and constipation, but also the number of visits and hospitalizations. 

In conclusion, *L. reuteri DSM17938* is effective, and may be recommended for breastfed infants with colic, while its role in formula-fed infants requires further study [119].

## 7. Safety of Probiotics in Pregnancy and Neonatal Period

The early supplementation of probiotics in the perinatal and postnatal periods seems to have a positive impact on future health of infants. In this article we have shown the beneficial effects of probiotics in preventing infections before and after delivery and atopy in children. However, to define the possible role of the early administration of probiotics on the development of the nervous system of newborns, future studies and randomized trials are required. The use of probiotics is usually considered safe, even in first months of life. Despite the large use of probiotics during pregnancy and perinatal period, there are few studies that tested their safety during this period. Development of infections or other adverse effects in adult patients after the use of probiotics are rarely reported and often involve immunocompromised patients [120,121]. Allen et al., in a randomized, double-blinded, placebo-controlled trial, have tested the possible adverse effects of the administration of a probiotics mixture to pregnant women and to their infants after the birth. In this study, none of the adverse effects was attributed to the supplementation of probiotics [122]. Moreover, Baldassarre et al., in a prospective, double-blinded, randomized, controlled trial, confirmed that the early administration of probiotics during pregnancy has no side effects in mothers or in infants [115]. In addition, Luoto et al. in a clinical trial, involving 256 pregnant women and their offspring, showed no side effects in mothers and children after the administration of *Lactobacillus rhamnosus* GG and *Bifidobacterium lactis Bb12* probiotics mixture [123]. In contrast, Kuitunen et al. have suggested that the early supplementation of probiotics negatively influences hematologic values in infants. In this trial, children supplemented with probiotics before and after the birth have significantly lower haemoglobin levels compared to the placebo group at 6th month of life. This effect is transient, and may be due to a potential inflammation of the intestinal mucosa probably caused by probiotics [124] (Table 4). Future studies are needed to confirm the total safety of the use of probiotics during pregnancy and in the early stages of life.

## 8. Conclusions

In the last 20 years, it has been shown that the constitution of the human microbiome is conditioned by multiple elements such as genetic heritage, prematurity, cesarean section, kind of infant nutrition, administration of probiotics or antibiotics, perinatal stressors, and infections. Further studies demonstrated that a normal intestinal microbiota takes part in the induction of the immune tolerance [125,126]. Alterations of the microbiota are associated with the development of many pathological states like infantile colic, inflammatory bowel disease, necrotizing enterocolitis, asthma, atopic diseases, celiac disease, diabetes, mood disorders, and autism spectrum disorders. Additional studies are needed to attest that probiotics could have a protective function against the onset and the progression of these diseases. Nowadays there is no standard recommendation for performing targeted supplementation in individual patients.

Studies suggest that the microbiota may influence immunologic and inflammatory systemic responses, and thus, modulate the onset of sensitization and allergy.

In 2015, the WAO guidelines on the prevention of allergies recommends using probiotics in: (a) pregnant women at high risk for having an allergic child; (b) women who breastfeed infants at high risk of developing allergies; and (c) infants at high risk of developing allergies [73].

These are the only recommendations by the Scientific Community for using probiotics as a preventive intervention for diseases during pregnancy and perinatal period, despite the many studies demonstrating clinical benefits from the administration of probiotics in pregnancy and the perinatal period. So, it is important to obtain more data about the exact composition of microbiomes and the alterations which occur in specific diseases. It is also important to underline that the security and effectiveness of findings attributable to one single probiotic product cannot be applied to other probiotic formulations, especially if the product is administered to patients such as pregnant women and newborns; this is a serious task, and therapeutic discomfort that multi-strain probiotic formulations lack generic names, because they are food supplements. Many clinicians and patients are unaware that deficiencies in the regulation of probiotics mean that the formulations sold under these medically-recognized brand names may no longer be the same as the original products on which the clinical efficacy and safety evidence is based. FAO/WHO guidelines for probiotics state that proper nomenclature and strain designation are required on a probiotic product. Without proper identification of the strains and/or of the clarification of the origin of the product such as manufacturing site, the clinical evidence is erroneously transferred from one product to another. This is the reason why limiting the information to probiotic genera/species is not the best choice [127], and more stringent quality control of probiotics is required [128].

## Figures and Tables

**Table 1:** Role of probiotics administration in the prevention of infection and preterm delivery during pregnancy.

Author, Year	Study Design	Study Population	Intervention Strain Dose (D) Start of Treatment (S) End of Treatment (E)	Placebo	Outcomes Evaluations	Follow-Up	Side Effects
Gille et al., 2016 [50]	Randomized, placebo-controlled, triple-blind, parallel group trial	320 pregnant women	*L. rhamnousus,* GR-1^®^ and *L. reuteri,* RC-14^®^ D: 1 × 10^9^ colony-forming unit (CFU) of each strain S: first trimester of pregnancy E: after 8 weeks of treatment	Indistinguishable placebo capsule	- Proportion of normal vaginal microbiota Main outcome: probiotics not improve the normal composition of vaginal microbiota compared to the placebo group	No available	Not observed
Olsen et al., 2010 [53]	Pilot randomised controlled trial	34 Group B streptococcus—positive pregnant women	*Lactobacillus rhamnosus* GR-1 (GR- 1) and *Lactobacillus fermentum/reuteri* RC-14 (RC-14) D: 1 × 10^8^ CFU viable strain S: 36 weeks of gestation E: for three weeks or until the birth	No probiotics in control group	- Incidence of vaginal Group B streptococcus colonization Main outcome: no significant difference in the incidence of GBS vaginal infections between the women supplemented with probiotics and the control group	6 months after delivery	Not observed
Ho et al., 2016 [54]	Prospective, double-blind randomized clinical trial	110 GBS-positive pregnant women	*L. rhamnosus* GR-1 and *L. reuteri* RC-14 D: 1 × 10^9^ CFU of both strain S: at 35 e 37 weeks of gestation E: at delivery	Indistinguishable placebo capsule	- Incidence of vaginal GBS colonization - Cause of admittance to the neonatal Unit Main outcome: Probiotics administration significantly reduced rectal and vaginal GBS colonization rate	No available	Not observed
Krauss-Silva Krauss-Silva et al., 2011 [57]	Prospective Double blind Randomized Controlled	664 pregnant women	*Lactobacillus rhamnosus* GR-1 and *Lactobacillus reuteri* RC-14 D: 2 × 10^6^ CFU of each strain S: 20 weeks of gestation E: at delivery	Indistinguishable placebo capsule	- Incidence of spontaneous preterm delivery - Neonatal morbidities Main outcome: no conclusive results on the efficacy of probiotics in the prevention of preterm birth	No available	adverse events minor and non-specific of probiotics use
Hantoushzadeh et al., 2012 [58]	Double-blind, placebo-controlled, parallel-group randomized clinical trial	310 pregnant women with symptomatic BV	Probiotic yogurt: *lactobacillus bulgaris, streptococcus thermophilus, probiotic lactobacillus, and bifidobacterium lactis* D: 100 g twice a day for one week S: third trimester of pregnancy E: for one week	Orally-administered clindamycin (300 mg twice a day for 1 week)	- BV cure rate after one week of treatment - Preterm birth, Premature rupture of membranes, pH decrease and recurrence Main outcome: reduction of vaginal pH in women supplemented with probiotics	Until delivery	Not observed

**Table 2:** Probiotics administration during pregnancy and after delivery in prevention of allergic disorders.

Author, Year	Study Design	Study Population	Intervention Strain Dose (D) Start of Treatment (S) End of Treatment (E)	Placebo	Outcomes Evaluations	Follow-Up	Side Effects
Dotterud et al., 2015 [64]	Randomized, double-blind trial	415 pregnant women	Probiotic milk: Biola ^®^ (Tine BA, Oslo, Norway), contained *Lactobacillus rhamnosus* GG (LGG), *Bifidobacterium animalis subsp. lactis* Bb-12 (Bb-12) and *L. acidophilus* La-5 (La-5). D: 5 × 10^10^ CFU of LGG and Bb-12, and 5 × 10^9^ colony-forming unit (CFU) of La-5 daily S: 4 weeks before the expected delivery date (to mothers) E: 3 weeks after delivery(to mothers during breastfeeding)	Indistinguishable placebo milk	- Development of atopic diseases in children (asthma, atopic dermatitis and allergic rhinoconjunctivitis) Main outcome: probiotics administration reduces the incidence of AD in children	24 months after delivery	Not observed
Enomoto et al., 2014 [65]	Open-trial study	166 pregnant women	*B. longum* BB536 [ATCC BAA-999] and *B. breve* M-16V [LMG 23729] D: two sachets, each containing approximately 5 × 10^9^ CFU of both probiotics S: 4 weeks before the expected delivery date (to mothers) E: 6 months after delivery(to infants)	The control group no received probiotics	- Development of allergic symptoms in children - Composition of faecal samples (mothers and infants) Main outcome: probiotics administration reduces the incidence of AD/eczema in children	36 months after delivery	Not observed
Wickens et al., 2018 [66]	Randomized placebo-controlled trial	423 pregnant women	*Lactobacillus rhamnosus* HN001 (HN001) D: 6 × 10^9^ CFU S: from 14–16 weeks gestation (to mothers) E: 6 months post-partum (to mothers during breast-feeding)	Indistinguishable placebo capsules	- Development of atopic diseases in children - Immunomodulatory factors in breast milk (TGF-β1, TGF-β2) Main outcome: probiotic supplementation not prevent infant eczema	12 months after delivery	Not observed
Ou et al., 2012 [67]	Prospective, double-blind, placebo-controlled clinical trial	191 pregnant women	*Lactobacillus GG;* ATCC 53103; D: 1 × 10^10^ CFU daily S: From the second trimester of pregnancy (to mothers) E: 6 months post-partum (to mothers during breastfeeding and to infants)	microcrystalline cellulose	- Development of allergic diseases in children. - Improvement of maternal allergic symptom score and plasma immune parameters Main outcome: probiotic supplementation not prevent infant allergic disease	36 months after delivery	Not observed
Rautava et al., 2012 [68]	Parallel, double-blind placebo-controlled trial	241 pregnant women	(1) *Lactobacillus rhamnosus LPR* and *Bifidobacterium longum BL999 (LPR+BL999)* (2) *L paracasei ST11* and *B longum BL999 (ST11+BL999)* D: 1 × 10^9^ CFU for each probiotic S: 2 months before delivery (to mothers) E: 2 months post-partum (to mother during breast-feeding)	Indistinguishable placebo	- Development of allergic diseases in children. Main outcome: probiotic supplementation prevents infant eczema	24 months after delivery	Not observed
Kim et al., 2010 [69]	Randomized, double-blind, placebo-controlled trial	112 pregnant women	*Bifidobacterium bifidum* BGN4, *B. lactis* AD011, and *Lactobacillus acidophilus* AD031 D: 1.6 × 10^9^ CFU for each probiotic S: 4–8 weeks before delivery (to mothers) E: until 6 months after delivery (to mothers during breastfeeding and to infants)	Indistinguishable powder	Assess the occurrence of eczema Main outcome: probiotics administration reduces the incidence of eczema in children	12 months after delivery	adverse events minor and non-specific of probiotics use
Niers et al., 2009 [70]	Double-blind, randomized, placebo-controlled trial	136 pregnant women	*Bifidobacterium bifidum, Bifidobacterium lactis,* and *Lactococcus lactis* D: 1 × 10^9^ CFU of each strain S: last 6 weeks of pregnancy (to mothers) E: 12 months after delivery (to infants)	Indistinguishable powder	- Development of allergic diseases in infants - Molecular analysis of fecal microbiota in infants - Cytokine analysis in infants Main outcome: probiotics administration reduces the incidence of eczema in children at 3rd month of life	24 months after delivery	Not observed
Simpson et al., 2015 [71]	Randomised controlled trial	415 pregnant women	Probiotic milk: *Lactobacillus rhamnosus* GG, *L. acidophilus* La-5 and *Bifidobacterium animalis* subsp. lactis Bb-12 D: 5 × 10^10^ CFU of *Lactobacillus rhamnosus* and *Bifidobacterium animalis* and 5 × 10^9^ CFU of L. acidophilus La-5 S: from 36 weeks gestation (to mothers) E: until 3 months postpartum (during breast-feeding)	Placebo milk	Development of allergic diseases in infants Main outcome: probiotics administration reduces the incidence of atopic dermatitis	6 years after delivery	Not observed

**Table 3:** Probiotics and functional gastrointestinal disorders.

Author, Year	Study Design	Study Population	Intervention Strain Dose (D) Start of Treatment (S) End of Treatment (E)	Placebo	Outcomes Evaluations	Follow-Up	Side Effects
Partty et al., 2013 [114]	Randomized Double-blind Prospective Follow up	159 women	*L. rhamnosus GG* (ATCC 53103) D: not available S: 4 weeks before expected delivery E: 6 months after delivery to child or to the mother if breast-feeding	Indistinguishable powder	Functional gastrointestinal disorders Main outcome: administration of *L. rhamnosus GG* (ATCC 53103) does not affect the appearance of functional gastrointestinal disorders later in childhood	13 years	Not observed
Baldassarre et al., 2016 [115]	Prospective Double-blind Randomized Controlled	66 women aged 18–44	Probiotic mixture: *L. paracasei* DSM 24733, *L. plantarum* DSM 24730, *L. acidophilus* DSM 24735, *L. delbrueckii* subsp. *bulgaricus* DSM 24734, *B. longum* DSM 24736, *B. breve* DSM 24732, *B. infantis* DSM 24737, *Streptococcus thermophilus* DSM 24731 D: 900 billion S: 4 weeks before expected delivery E: 4 weeks after delivery	Indistinguishable powder	- cytokine profile and secretory IgA in breast milk - lactoferrin and sIgA levels in stool samples of newborns - newborn gastrointestinal symptoms - neonatal growth pattern Main outcome: maternal supplementation with probiotic modulates breast milk cytokines pattern in newborns and improves gastrointestinal functional symptoms	4 weeks after delivery	Not observed

**Table 4:** Safety of probiotics in pregnancy and neonatal period.

Author, Year	Study Design	Study Population	Intervention Strain Dose (D) Start of Treatment (S) End of Treatment (E)	Placebo	Outcomes Evaluations	Follow-Up	Side Effects
Luoto et al., 2010 [123]	Double-blind, placebo-controlled study	256 pregnant women	*Lactobacillus rhamnosus* GG and *Bifidobacterium lactis* Bb12 D: 10^10^ colony-forming unit (CFU)/d each S: first trimester of pregnancy (to mothers) E: to the end of exclusive breastfeeding	Indistinguishable placebo capsule	- Safety and efficacy of probiotic in mothers and infants Main outcome: probiotics supplementation reduced the frequency of gestational diabetes mellitus with a normal duration of pregnancies and no adverse events in mothers or children	24 months after delivery	Not observed
Allen et al., 2010 [122]	Randomized, double-blinded, placebo-controlled trial	454 pregnant women	Two strains of lactobacilli *(Lactobacillus salivarius CUL61 and Lactobacillus paracasei CUL08*) and bifidobacteria *(Bifidobacterium animalis subsp. lactis CUL34 and Bifidobacterium bifidum CUL20)* D: a total of 1 × 10^10^ CFU/d S: last month of pregnancy (to mother) E: until 6 months of life (to infants)	Indistinguishable placebo capsule	- Symptoms and adverse effects in mother and infants Main outcome: No side effects were attributed to probiotics supplementation	24 months after delivery	Not observed
Kuitunen et al., 2009 [124]	Prospective randomized controlled trial	1223 pregnant women	*Lactobacillus rhamnosus GG (ATCC 53103)* 5 × 10^9^ CFU, *L rhamnosus LC705* *(DSM 7061)* 5 × 10^9^ CFU*, Bifidobacterium breve Bb99 (DSM* *13692) 2 × 10^8^* CFU, and *Propionibacterium freudenreichii ssp* *shermanii JS (DSM 7076)* 2 × 10^9^ CFU S: 4 weeks before delivery (to mothers) E: until 6 months of life (to infants)	Indistinguishable placebo	- Safety and efficacy of probiotic in mothers and infants - Blood and faecal samples taken from children Main outcome: Infants in the probiotic group have lower haemoglobin levels compared to the placebo group at 6th month of life	24 months after delivery	lower haemoglobin levels at 6th month of life
